# Crosstalk between vimentin and keratins in viral infection: Implications across the viral life cycle

**DOI:** 10.1080/21505594.2026.2646692

**Published:** 2026-03-23

**Authors:** Xiaolong Lu, Xiaoquan Wang, Xiufan Liu, Xiaowen Liu

**Affiliations:** a Key Laboratory of Avian Bioproducts Development, Ministry of Agriculture and Rural Affairs, College of Veterinary Medicine, Yangzhou University, Yangzhou, PR China; b Jiangsu Co-Innovation Center for Prevention and Control of Important Animal Infectious Diseases and Zoonoses, Yangzhou University, Yangzhou, PR China; c Jiangsu Interdisciplinary Center for Zoonoses and Biosafety, Yangzhou University, Yangzhou, PR China; d Jiangsu Key Laboratory of Zoonosis, Yangzhou University, Yangzhou, PR China; e Joint International Research Laboratory of Agriculture and Agri-Product Safety of Ministry of Education of China, Yangzhou University, Yangzhou, PR China

**Keywords:** Intermediate filaments, vimentin, keratin, viral infection, antiviral intervention

## Abstract

Intermediate filaments (IFs) have long been regarded as a static scaffold responsible for maintaining cellular structure and integrity. However, recent studies have revealed that IFs, particularly vimentin and keratin, exert a profound and versatile influence on viral infection. In this narrative review, we summarize how these IFs influence multiple stages of the viral life cycle, including attachment/entry, replication, intracellular trafficking, assembly, and egress. We further discuss their contributions to cell-to-cell spread, host immune regulation, and oncogenic processes. Collectively, these findings illustrate how viruses exploit or remodel the IF network to facilitate propagation, and highlight IF – virus interfaces as potential targets for antiviral intervention.

## Introduction

The successful infection of a virus depends on its ability to precisely hijack and modify host cells [1]. The cytoskeletal system, composed of actin filaments, microtubules, and intermediate filaments (IFs), is a primary target for viral hijacking within the host cell [2,3]. IFs constitute a large, cell type – specific protein superfamily that complements actin and microtubules by providing stress resistance and long-range intracellular connectivity, while also serving as signaling platforms that integrate mechanical and biochemical cues [4,5]. Based on sequence homology and assembly properties, cytoskeletal IF proteins are commonly classified into multiple types: type I and II IFs are acidic and basic keratins, respectively; type III includes vimentin and related proteins (e.g. desmin, GFAP, peripherin); type IV comprises neuron-associated IFs (e.g. neurofilaments and α-internexin); type V corresponds to nuclear lamins; type VI group consists of lens-specific IFs, CP49/phakinin and filen [6,7].

**Figure 1.**
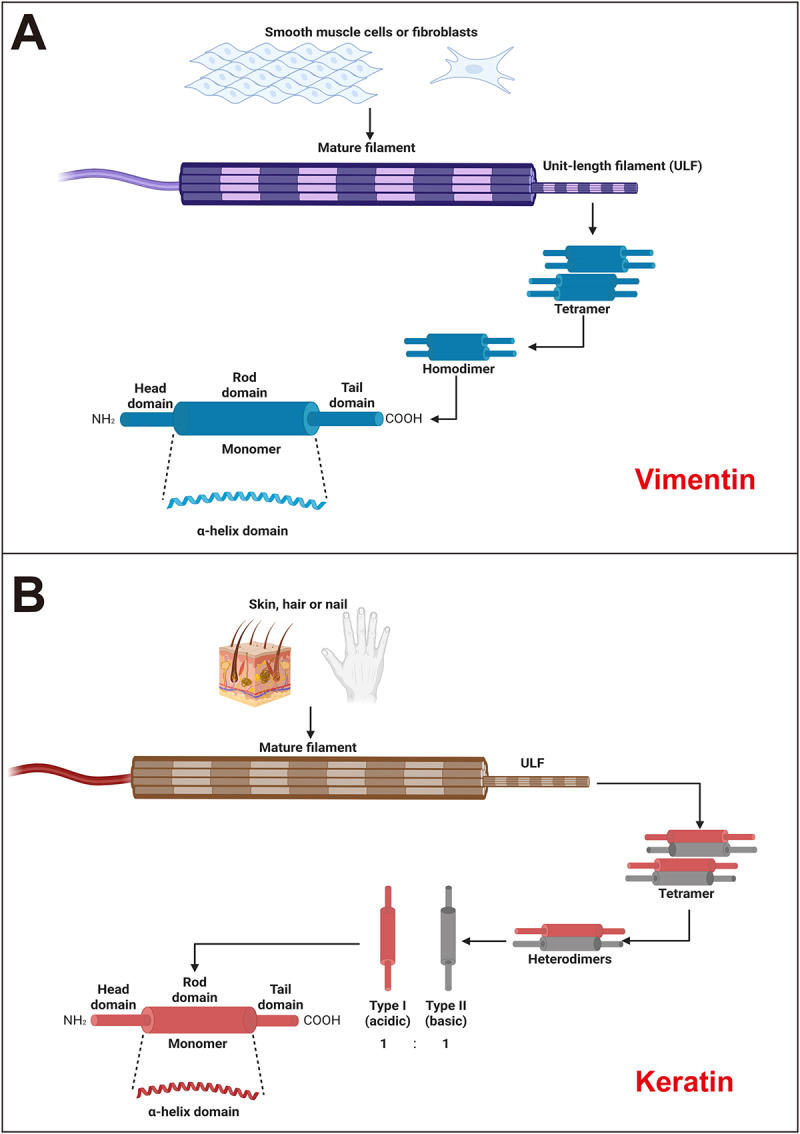
Distinct assembly mechanisms of vimentin and keratin intermediate filaments. (A) Vimentin, a type iii IF found in fibroblasts and smooth muscle cells, polymerizes from monomers into parallel, in-register coiled-coil dimers, which form antiparallel tetramers. Vimentin tetramers serve as soluble subunits that laterally compact and longitudinally anneal to generate mature, unit-length filaments. (B) Keratins, the major IFs of skin, hair, and nails, are obligate heteropolymers. Their assembly initiates with the heterodimerization of type I and type ii polypeptides, which then associate to form staggered, antiparallel tetramers. Keratin tetramers laterally associate and longitudinally anneal to form mature, unit-length filaments. The schematic representation is created with Biorender.com.

IFs are the most complex and stable type of cytoskeleton. In epithelial cells, the IF network is primarily composed of keratins. Conversely, vimentin is the main IF protein found in mesenchymal cells [8,9]. Vimentin, a type III IF protein, polymerizes from monomers into 10 nm filaments. This assembly proceeds through the formation of parallel, in-register coiled-coil dimers and subsequent antiparallel tetramers, which are the fundamental soluble subunits. A hallmark of the monomer is the central α-helical rod domain, which is bordered by intrinsically disordered head and tail regions [10–12] (Figure 1(A)). By contrast, keratins are obligate heteropolymers. The monomer is characterized by a central α-helical rod domain flanked by intrinsically disordered head and tail regions. Their assembly is initiated by the precise 1:1 pairing of type I (acidic) and type II (basic) polypeptides. This interaction forms coiled-coil heterodimers, which subsequently associate into staggered antiparallel tetramers [13,14]. These tetramers then laterally compact and longitudinally anneal to form unit-length filaments that ultimately mature into robust intermediate filaments, exhibiting extensive type-specific diversification across epithelial tissues [15–17] (Figure 1(B)). Traditionally, these proteins were regarded primarily as structural elements, responsible for maintaining cell shape and resisting mechanical stress [18,19].

However, this intracellular structural view is now incomplete. In addition to their filamentous cytoplasmic networks, both vimentin and selected keratins can also be detected at the cell surface or in the extracellular milieu, indicating that IF proteins are not restricted to the cytoskeleton but can also function at the host – environment interface [20–23]. Among IF proteins, extracellular and cell-surface vimentin have been characterized most extensively. In addition to its canonical cytoplasmic filament network, vimentin can be actively released into the extracellular space by activated macrophages and through type III unconventional secretion pathways, and it can also appear as a bona fide cell-surface protein [20,21,24]. Extracellular vimentin is not merely a by-product of cell damage, but can function as an immunologically active molecule, including as a damage-associated molecular pattern (DAMP) and regulator of neutrophil responses, and may also become exposed during inflammatory processes such as NETosis [25]. Although less well studied, keratins can likewise be detected outside the canonical cytoplasmic IF network. Cell-surface expression has been reported for specific keratins, particularly keratin 8 (KRT8) and keratin 1 (KRT1), in stressed or transformed cells, and keratins can also become exposed on apoptotic or necrotic cells, where they participate in immune recognition [22,23,26,27]. Compared with vimentin, however, the mechanisms underlying keratin externalization remain less clearly defined.

However, more and more evidence shows that these two IF proteins act as dynamic signaling platforms. They are deeply involved in many physiological and pathological processes, such as cell migration, signal transduction, and stress responses [28]. Importantly, recognition of extracellular and cell-surface pools of vimentin and keratin substantially broadens the conceptual framework of IF biology. These proteins should now be considered not only intracellular scaffolds but also context-dependent extracellular effectors and surface-accessible ligands/receptors that may shape pathogen attachment, host sensing, inflammatory signaling, and tissue tropism at the earliest stages of infection. In the field of viral infection, the roles of vimentin and keratin have changed from simple structural support to key regulatory factors. Their central importance in the interaction between virus and host is becoming increasingly clear. Although several vimentin-focused reviews have emerged in recent years [29,30], a comprehensive synthesis that jointly integrates vimentin and keratin across the viral life cycle remains lacking. Accordingly, this narrative review is based on the latest research and will critically synthesize the complex functions of vimentin and keratin during the viral life cycle, including virus entry, replication, transport, assembly, release, spread, and immune regulation. Finally, we will look at their future potential as targets for antiviral drugs.

## Contribution of intermediate filaments to the early stages of viral infection

**Figure 2.**
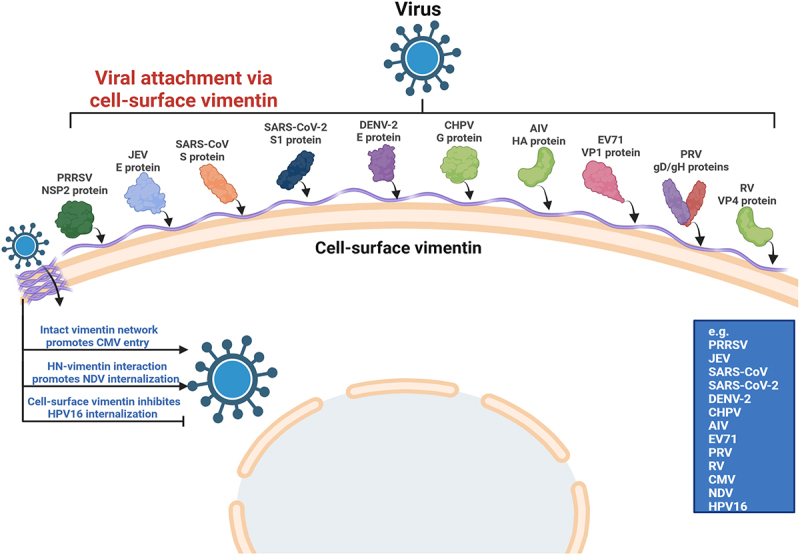
Cell surface vimentin as a critical mediator for viral entry. As a key component on the cell surface, vimentin can function as an attachment point, receptor, or co-receptor for diverse viruses. For PRRSV, vimentin interacts with NSP2 to form a receptor complex that mediates adsorption and confers susceptibility to non-permissive cells. JEV binds directly to vimentin via its E protein; this interaction is enhanced by D2R signaling and contributes to the virus’s cellular tropism. SARS-CoV and SARS-CoV-2 spike proteins also engage vimentin, which acts synergistically with ACE2 to facilitate entry, a process inhibitable by antibody blockade or gene knockdown. Other RNA viruses, including DENV-2, CHPV, H9N2 AIV, EV71, and NDV, similarly exploit vimentin through interactions with their respective envelope or capsid proteins (e, G, HA, VP1, HN) to promote adsorption and internalization. Vimentin also facilitates the entry of DNA viruses such as PRV, CMV, and HBV. PRV binds the rod domain of vimentin via its gD/gH proteins. In contrast to its typical pro-viral role, surface vimentin acts as a restrictive factor for HPV16, impeding viral internalization after attachment and inhibiting infection. The schematic representation is created with Biorender.com.

The invasion of host cells by viruses begins with their precise recognition and binding to receptors on the cell surface [31]. Current evidence indicates that intermediate filaments, especially vimentin and, to a lesser extent, keratins, are not merely structural components at this stage, but active determinants of viral attachment and entry. Studies have found that IF proteins located on the cell surface can serve as attachment factors, receptors, or co-receptors for various viruses, thereby influencing viral adsorption and internalization. Among them, cell-surface vimentin is the most extensively studied and appears to function as a broadly used but virus-dependent entry factor. In the context of RNA virus infection, cell-surface vimentin has been identified as a host factor that facilitates porcine reproductive and respiratory syndrome virus (PRRSV) infection. It functions as a receptor for PRRSV and interacts with the viral NSP2 protein to form a complex that promotes viral adsorption. Notably, it also serves as a core component of the receptor complex and can confer susceptibility to otherwise non-susceptible cells [32–34]. Similarly, the E protein of Japanese encephalitis virus (JEV) binds cell-surface vimentin as a key receptor, and antibody blockade markedly inhibits infection [35,36]. This role is further modulated by host signaling: D2R stimulation activates phospholipase C (PLC), which increases cell-surface vimentin expression and thereby enhances JEV entry [37]. The cell type-specific distribution of cell-surface vimentin – high in neural progenitor and glial cells but absent in mature neurons – has also been linked to the tropism of JEV [38]. A comparable pattern has emerged for coronaviruses, supporting the idea that cell-surface vimentin often acts as an accessory entry factor that cooperates with canonical receptors. In SARS-CoV infection, the spike protein binds vimentin and promotes its surface accumulation, whereas antibody blockade or genetic inhibition of cell-surface vimentin reduces viral binding and entry [39]. In SARS-CoV-2 infection, cell-surface vimentin likewise functions as an attachment factor or co-receptor. It binds the spike protein, particularly the S1 subunit, and cooperates with ACE2 to promote viral entry in multiple cell types, including endothelial cells [40–43]. The cell-surface vimentin – spike interaction may occur at specialized surface regions such as cilia, further suggesting that cell-surface vimentin helps organize local docking platforms for viral attachment [44]. This entry-promoting role is not restricted to PRRSV, JEV, or coronaviruses. The EDIII domain of the Dengue virus 2 (DENV-2) E protein binds cell-surface vimentin to facilitate adsorption [45]. On neuronal cells, Chandipura virus (CHPV) uses cell-surface vimentin as a co-receptor through its glycoprotein G [46]. H9N2 avian influenza virus (AIV), Enterovirus 71 (EV71), and Newcastle disease virus (NDV) also depend on cell-surface vimentin during early infection [47–51]. DNA viruses exploit the same principle: Pseudorabies virus (PRV) binds the rod domain of cell-surface vimentin through conserved sites in its gD and gH proteins [52], Cytomegalovirus (CMV) entry is facilitated by the vimentin network [53,54], and Hepatitis B virus (HBV) requires vimentin for endocytic entry [55]. Cell-surface vimentin also interacts with Rotavirus (RV) VP4 together with ACTR2 to promote early viral infection [56]. Overall, these findings support the view that cell-surface vimentin serves as a versatile host entry platform exploited by phylogenetically diverse viruses. These findings suggest that cell-surface vimentin is not a virus-specific receptor in a narrow sense, but a versatile host entry platform exploited by diverse viruses. However, the role of vimentin in entry is not uniformly pro-viral. In Human papillomavirus 16 (HPV16) infection, cell-surface vimentin restricts viral internalization after attachment, thereby inhibiting infection [57,58] (Figure 2). Thus, vimentin is better viewed as a multifunctional entry determinant whose effect depends on the entry strategy of each virus.

**Figure 3.**
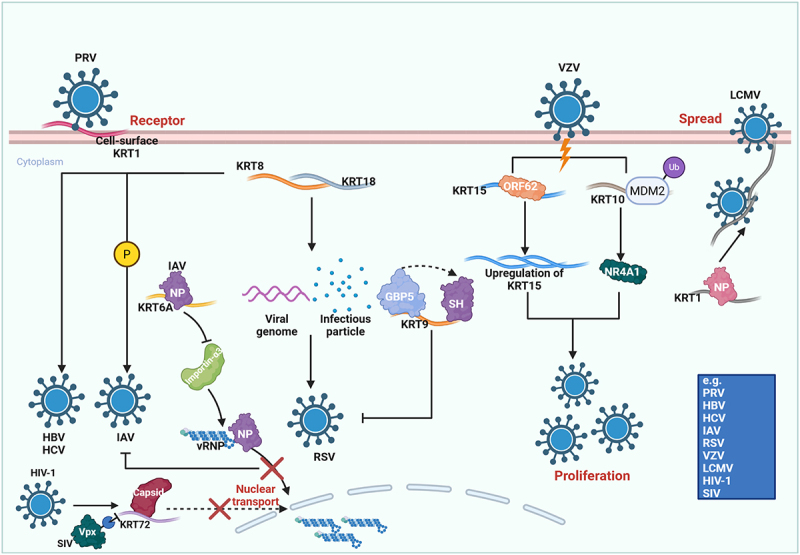
Keratins: multifaceted regulators of the viral life cycle. Keratins orchestrate a complex relationship with viruses throughout the infection process. Entry: Keratins can act as essential attachment factors or receptors, exemplified by KRT1 for PRV, where its blockade inhibits infection. Replication: their role is dualistic. While KRT8, KRT15, and KRT18 promote the replication of diverse viruses (HBV, HCV, RSV, VZV), others like KRT6A, KRT9, and KRT10 act as intrinsic antiviral factors by targeting viral proteins (e.g. IAV NP, RSV sh) or modulating host pathways (e.g. NR4A1). Trafficking: Keratins regulate intracellular viral transport; KRT6A impedes nuclear import of IAV vRnps, while KRT72 restricts HIV-1 capsid trafficking, a mechanism counteracted by SIV vpx. Transmission: finally, keratins can facilitate cell-to-cell spread, as LCMV exploits KRT1 to enhance cytoskeletal stability and cell adhesion. The schematic representation is created with Biorender.com.

Compared with vimentin, keratins have been studied less extensively as entry factors, but available data indicate that they also participate in early virus – host interactions. Cell-surface KRT1 has been identified as a key receptor for PRV entry, and both antibody blockade and gene knockout markedly reduce cellular susceptibility [59]. The epithelial-surface keratins contribute to epithelial barrier integrity, indicating that their role at the entry stage is intrinsically dual: they may facilitate viral attachment in some settings while also limiting tissue invasion through maintenance of epithelial structure [60] (Figure 3 and Table S1).

## The central role of intermediate filaments in viral replication process

Upon entry into the host cell, the virus hijacks cellular machinery and resources to initiate genome replication and viral protein synthesis. At this stage, vimentin emerges as a dynamic scaffold that is repeatedly remodeled to support replication, rather than as a passive cytoskeletal bystander. In several systems, an intact vimentin network is essential for efficient viral replication. For example, the synthesis of EV non-structural proteins such as 2A and 3D depends on intact vimentin, and pharmacological disruption of vimentin inhibits Vaccinia virus (VACV) replication [61,62]. A recurrent theme is that many viruses induce vimentin reorganization into perinuclear, granular, or cage-like structures that support replication complexes. JEV activates a CDK1–PLK1 kinase cascade that phosphorylates vimentin, causing its fragmentation into granules that traffic to the endoplasmic reticulum and form a cage-like structure supporting replication [63]. NDV uses a similar strategy by activating MLC kinase to induce vimentin reorganization into a cage-like structure [64]. PRRSV activates CaMKIIγ to phosphorylate vimentin, which assembles a cage around its replication complex to enhance viral efficiency, while Porcine circovirus type 2 (PCV2) utilizes the same pathway to alter vimentin’s cellular distribution for replication, revealing that CaMKIIγ-mediated vimentin phosphorylation is a key replication strategy shared by multiple viruses [65,66]. DENV-2 also induces structural rearrangement of vimentin through ROCK-dependent phosphorylation and perinuclear reorganization [67,68], whereas Zika virus (ZIKV) and Chikungunya virus (CHIKV) likewise induce vimentin cages around replication factories [69,70]. Although the upstream kinases differ, the underlying strategy is similar: viruses exploit vimentin phosphorylation and spatial remodeling to generate a protected replication microenvironment.

**Figure 4.**
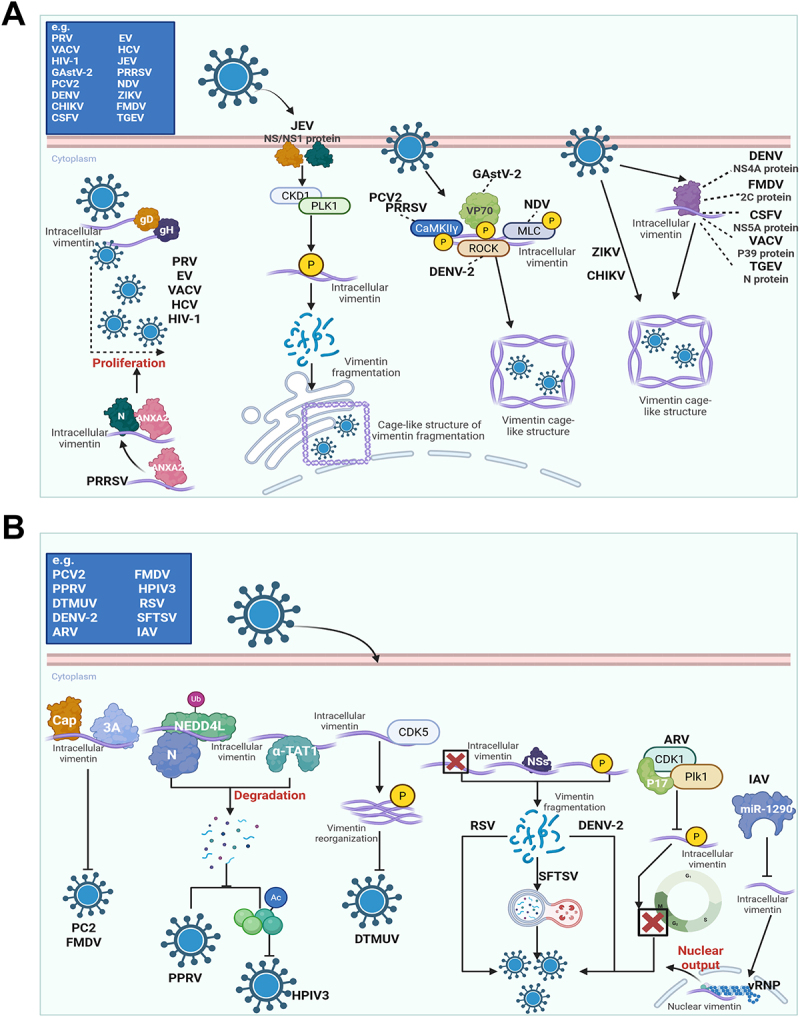
The dual functionality of vimentin in viral replication. (A) Pro-viral functions of vimentin. An intact vimentin network is required for efficient replication of EV and VACV. Many viruses – including JEV, NDV, PRRSV, PCV2, DENV-2, ZIKV, and CHIKV – hijack host kinases to phosphorylate vimentin, reorganizing it into cage-like structures that protect replication sites. Vimentin is also essential for HCV and HIV-1 replication. Numerous viral proteins directly bind vimentin to exploit its scaffolding function, such as VP70 (GAstV-2), gD/gH (PRV), NS4A (DENV), 2C (FMDV), NS5A (CSFV), P39 (VACV), and N (TGEV). For PRRSV, vimentin first binds ANXA2 to promote interaction with the viral N protein. (B) Vimentin as an antiviral factor and viral countermeasures. Vimentin inhibits viruses by directly binding proteins like Cap (PCV2) and 3A (FMDV), recruiting the E3 ligase NEDD4L to degrade PPRSV N protein, or suppressing inclusion body formation in HPIV3. DTMUV replication is inhibited by vimentin phosphorylation via CDK5. To overcome this, viruses have evolved strategies: RSV and SFTSV degrade vimentin; DENV-2 induces its network disassembly; and ARV and IAV manipulate its expression/phosphorylation to disrupt the cell cycle and vRNP trafficking, respectively. The schematic representation is created with Biorender.com.

In addition to network remodeling, many viruses directly engage vimentin through specific viral proteins. The VP70 protein of Goose astrovirus type 2 (GAstV-2) interacts with vimentin to induce its phosphorylation and reorganization into a complex that encapsulates viral RNA [71]. As noted above, PRV gD and gH interact with the vimentin rod domain, and in PRRSV infection, vimentin first binds ANXA2 and then promotes its interaction with the viral N protein, thereby enhancing replication [72]. Other viral proteins reported to bind vimentin include DENV NS4A [73], Foot-and-mouth disease virus (FMDV) 2C [74], Classical swine fever virus (CSFV) NS5A [75], VACV P39 [76], and Transmissible gastroenteritis virus (TGEV) N [77]. These examples indicate that vimentin supports replication through both structural remodeling and direct virus – host protein interaction. In addition, vimentin has also been reported to be required for efficient replication of Hepatitis C virus (HCV) and Human immunodeficiency virus type 1 (HIV-1) [78,79]. Notably, the contribution of vimentin to HCV infection may be dose-dependent: when highly expressed, vimentin can exert an inhibitory effect by accelerating degradation of the viral core protein [80] (Figure 4(A)).

Importantly, the relationship between vimentin and viral replication is not one-sided. Vimentin can also function as an antiviral factor under specific conditions. It directly binds the PCV2 Cap protein or the FMDV 3A protein to inhibit replication [81,82]. It can recruit the E3 ubiquitin ligase NEDD4L to promote degradation of the Peste des petits ruminant virus (PPRV) N protein [83], and it inhibits Human parainfluenza virus type 3 (HPIV3) inclusion body formation by promoting α-TAT1 degradation and reducing α-tubulin acetylation [84]. In Duck tembusu virus (DTMUV) infection, CDK5-mediated vimentin phosphorylation and reorganization are associated with inhibition of viral replication [85]. Vimentin also exerts antiviral effects through maintenance of intracellular cholesterol homeostasis [86]. Thus, vimentin is better regarded as a contested platform during replication, one that can be exploited by viruses but can also be mobilized by the host. Consistent with this, several viruses have evolved strategies to counteract vimentin. Respiratory syncytial virus (RSV) and Severe fever with thrombocytopenia syndrome virus (SFTSV) promote vimentin degradation [87,88], DENV-2 induces its phosphorylation and solubilization [89], and Avian reovirus (ARV) and Influenza A virus (IAV) manipulate its expression or phosphorylation to favor viral replication and vRNP trafficking [90,91] (Figure 4(B)).

Keratins also display clear functional duality during viral replication, but their effects appear more dependent on the individual keratin member. Keratin 18 (KRT18) and KRT8 are required for RSV replication [92]; KRT8 also promotes Hepatitis B virus (HBV) and HCV replication [93,94]; phosphorylation of KRT8 enhances H1N1 subtype IAV replication [95]; and keratin 15 (KRT15) interacts with the Varicella-zoster virus (VZV) ORF62 protein, leading to the upregulation of its expression and subsequent promotion of viral replication [96]. In contrast, keratin 9 (KRT9) acts as an antiviral factor against RSV by facilitating GBP5-dependent capture and secretion of the viral SH protein [97]. Likewise, VZV promotes replication by inducing MDM2-mediated degradation of keratin 10 (KRT10) [98], and keratin 6A (KRT6A) inhibits IAV replication by binding NP, disrupting vRNP integrity, and blocking nuclear import [99] (Figure 3). Therefore, keratins cannot be generalized as simply pro-viral or antiviral; their effects are strongly member- and virus-specific.

## Intermediate filaments as a critical platform for viral transport and assembly

**Figure 5.**
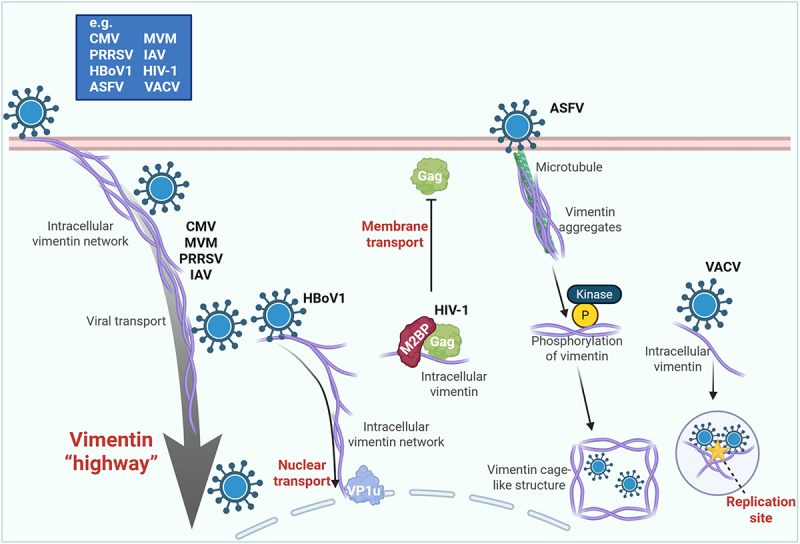
Vimentin orchestrates viral intracellular transport and assembly. Vimentin-mediated viral transport. Vimentin acts as a “highway” to facilitate the intracellular trafficking of diverse viruses. It promotes the transport of CMV and the endosomal trafficking of MVM. It collaborates with other cytoskeletal elements to transport PRRSV to the perinuclear region and accelerates IAV trafficking from early to late endosomes, enhancing membrane fusion. The interaction between vimentin and HBoV1 VP1u mediates the nuclear import of VP1u, boosting infection. Conversely, the host can exploit vimentin for defense; the protein M2BP anchors HIV-1 gag to vimentin filaments, blocking its transport to the plasma membrane and inhibiting virion production. Vimentin as a scaffold for viral assembly. Vimentin is frequently co-opted as a dynamic scaffold for viral assembly. During ASFV infection, vimentin is aggregated via microtubules and phosphorylated to form a perinuclear cage that concentrates viral proteins. Similarly, VACV infection recruits vimentin to viral replication sites to facilitate assembly. The schematic representation is created with Biorender.com.

For productive infection, viral components must be efficiently transported to appropriate intracellular sites and assembled into new virions. Current evidence suggests that vimentin contributes to these processes mainly by organizing intracellular trafficking and supporting localized assembly sites. It can act as a “highway” to actively promote infection. The vimentin network facilitates transport of CMV [53], promotes endosomal trafficking of Minute virus of mice (MVM) [100], collaborates with other cytoskeletal structures to assist the transport of PRRSV to the perinuclear region [101], and accelerates the trafficking of IAV from early to late endosomes, thereby promoting viral membrane fusion and genome release [102,103]. In addition, interaction between vimentin and the Human bocavirus 1 (HBoV1) VP1u protein enhances nuclear transport of VP1u and ultimately promotes infection [104]. These observations support the view that vimentin forms part of the intracellular architecture that coordinates viral movement. At the same time, this network can also be used for restriction. The host protein M2BP can capture the HIV-1 Gag protein and anchor it onto vimentin filaments, thereby blocking its transport to the plasma membrane and inhibiting virion production [105]. Thus, as in replication, the role of vimentin in transport is bidirectional. Vimentin also contributes to viral assembly. During African swine fever virus (ASFV) infection, it is reorganized into a perinuclear cage that concentrates viral proteins for assembly [106], and VACV recruits vimentin to the vicinity of viral replication sites to facilitate assembly [76] (Figure 5).

Compared with vimentin, the role of keratins in viral transport has been less extensively characterized and appears mainly restrictive. KRT6A impairs IAV infection by interfering with NP interaction with importin α3, thereby blocking nuclear import [99]. Keratin 72 (KRT72) likewise restricts HIV-1 by hindering capsid transport toward the nucleus, although Simian immunodeficiency virus (SIV) Vpx can counteract this defense by targeting KRT72 for degradation [107] (Figure 3). Whether keratins also make broader contributions to viral assembly remains unclear.

## The complex roles of intermediate filaments in viral release and spread

**Figure 6.**
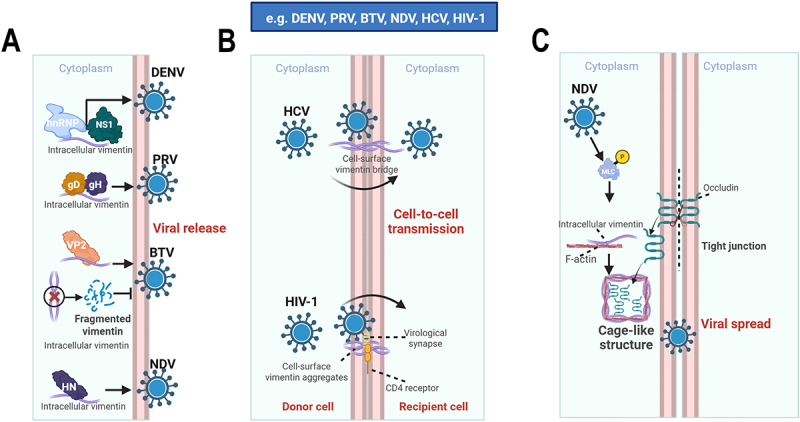
Vimentin-mediated viral release and cell-to-cell transmission. (A) Vimentin in facilitating viral release. Vimentin is critical for the release of diverse viruses. NDV HN protein engages vimentin to facilitate release. PRV promotes its release via gD and gH protein interaction with vimentin. ZIKV also requires vimentin for efficient release. For BTV, the outer capsid protein VP2 binds vimentin, and disrupting this interaction or the vimentin network inhibits release. During DENV infection, vimentin forms complexes with host hnRnps and the viral NS1 protein, promoting virus release. (B) Vimentin in cell-to-cell transmission and barrier disruption. Vimentin is crucial for direct viral spread between cells. HCV utilizes vimentin-containing cell bridges for directional transfer, mediated by the E1 protein. HIV-1 recruits vimentin to virological synapses to promote CD4 receptor clustering. (C) Viruses disrupt tissue barriers to accelerate spread. NDV induces reorganization of vimentin and F-actin, leading to the degradation of tight junction proteins and disrupting the cellular barrier. This enhances NDV cell-to-cell spread. The schematic representation is created with Biorender.com.

After completing replication, viruses need to be effectively released and spread to new cells or hosts. Although this phase has been studied less extensively than entry or replication, available evidence indicates that intermediate filaments influence both virion release and cell-to-cell spread. For vimentin, several studies support a role in viral egress. NDV engages vimentin through its HN protein to promote release [51]. PRV promotes its release by interacting with the rod domain of vimentin through its gD and gH proteins [52]. In addition to promoting efficient ZIKV replication, vimentin is also crucial for viral release, as its disruption severely hinders the process [69]. Release of Bluetongue virus (BTV) and DENV also depends on interactions with vimentin: the BTV outer capsid protein VP2 binds vimentin through residues glycine 70 and valine 72 [108], whereas DENV NS1 and host hnRNP-containing complexes are associated with vimentin during release [109] (Figure 6(A)). These studies suggest that the proviral functions of vimentin often extend throughout infection, from entry to late-stage egress.

Vimentin also facilitates direct intercellular spread. HCV exploits vimentin-containing cell bridges for directional transfer of viral components between adjacent cells, mediated by interaction between the HCV E1 protein and the vimentin head domain [110,111]. HIV-1 similarly recruits vimentin during formation of virological synapses, where it promotes CD4 clustering and supports cell-to-cell transmission [112] (Figure 6(B)). Thus, vimentin contributes not only to intracellular infection dynamics but also to intercellular dissemination. A further layer of complexity is that viruses can accelerate tissue spread by remodeling IF-dependent barrier structures. In NDV infection, reorganization of vimentin and F-actin induces degradation of tight-junction proteins such as OCLN and ZO-1, thereby weakening epithelial barrier integrity and favoring viral dissemination [64] (Figure 6(C)). This illustrates that IF remodeling can promote spread not only by acting on virions themselves, but also by dismantling host tissue barriers.

Evidence for keratin involvement in viral spread remains limited but is nonetheless suggestive. Lymphocytic choriomeningitis virus (LCMV) binds KRT1 through its NP protein, stabilizing the cytoskeleton and reinforcing cell – cell adhesion, which in turn promotes cell-to-cell spread [113] (Figure 3). At present, however, the contribution of keratins to release and dissemination remains much less defined than that of vimentin, highlighting an important gap in the field.

## The divergent roles of intermediate filaments in antiviral immunity and cancer development

IFs are not only exploited by viruses but also participate in host immune regulation. An important concept emerging from recent studies is that IF proteins regulate antiviral signaling in addition to their structural roles. Studies have found that virus-induced vimentin can inhibit the production of type I interferons by binding to and interfering with TBK1 and IKKε, thereby blocking IRF3 activation and helping the virus evade immune surveillance [114]. In Treg cells, vimentin anchors the IL-18 receptor and inhibits amphiregulin production, thereby impairing tissue repair after viral pneumonia [115]. These findings identify vimentin as a signaling hub that can be subverted to weaken antiviral defense and tissue recovery.

Conversely, IFs can also contribute to antiviral restriction. The interferon-induced protein MXB, for instance, achieves a broad-spectrum antiviral effect by recognizing vimentin and recruiting the kinase AKT to phosphorylate it. This disrupts the vimentin network, thereby blocking the transport of various viral protein complexes [116]. Keratins such as KRT9 and KRT72 likewise participate in host defense [97,107]. Furthermore, studies showing that high-risk HPV suppresses CD8+ T cell immune responses by overexpressing keratin 17 (KRT17) [117], while VZV promotes its own replication by degrading keratin KRT10 [98], further supports the importance of keratins in immune homeostasis.

The relevance of IFs extends beyond infection to cancer biology, particularly in the context of chronic infection, inflammation, and tissue remodeling. Vimentin is a canonical regulator of epithelial-mesenchymal transition (EMT), migration, and metastasis [118]. For example, the HBV HBx protein promotes hepatocellular carcinoma development by downregulating LINC01010 and thereby enhancing vimentin-dependent proliferation and migration [119]. Extracellular vimentin also contributes to communication between endothelial and immune cells within the tumor microenvironment [120]. Keratins, meanwhile, function not only as diagnostic markers but also as active regulators of tumor progression, as illustrated by keratin 7 (KRT7) and KRT17 [121–124]. Together, these observations indicate that IF proteins connect viral infection, immune dysregulation, and cancer progression through shared structural and signaling mechanisms.

## Conclusions and perspectives

In summary, vimentin and keratin play dynamic roles throughout the viral life cycle that extend far beyond structural support. Rather than acting as passive cytoskeletal components, they should be regarded as multifunctional host determinants that shape viral entry, replication, transport, assembly, release, spread, and immune responses. Overall, vimentin is more frequently exploited by viruses, whereas keratins display greater functional heterogeneity and often act in a member-specific manner. To facilitate the understanding of IF-mediated regulation during viral infection, we summarized the relevant findings in Supplementary Tables S2–S4.

This mechanistic diversity has important translational implications. First, the recurrent involvement of vimentin in viral attachment and entry supports vimentin-directed approaches as broad-spectrum entry interference strategies, including antibody-based blockade or competitive decoys that disrupt virion binding to surface-exposed vimentin [36,55,125]. Second, because many viruses rely on vimentin remodeling to build replication-competent microenvironments, targeting critical virus – vimentin interfaces or the host kinase circuits that drive vimentin phosphorylation and reorganization (e.g. ROCK and CDK/PLK-related pathways) may provide a rational means to suppress replication without directly targeting viral enzymes [63,68]. Third, given the dual roles of vimentin in innate immune signaling, therapeutic modulation of IF-linked immune regulation may help rebalance antiviral responses and limit immunopathology in selected settings [114,115]. Beyond intervention, IFs also carry biomarker potential: infection-associated changes in IF abundance, post-translational modification, or network architecture often correlate with tissue injury, barrier dysfunction, and disease severity, suggesting that IF remodeling signatures could inform risk stratification and treatment monitoring [126–129].

Despite rapid progress, several key issues remain unresolved. Knowledge of non-vimentin IFs, especially keratins, remains limited; most findings still rely heavily on *in vitro* systems; and the interplay among different IF proteins during infection is poorly understood. Future studies should therefore move beyond descriptive cataloging and focus more on shared mechanisms, virus-specific differences, and coordinated regulation within IF networks *in vivo*.

## Supplementary Material

Table S1.docx

Table S3.docx

Table S4.docx

Table S2.docx

## Data sharing statement

Data sharing is not applicable to this article as no new data were created or analyzed in this study.
