# Supplementary material for: Crosstalk between vimentin and keratins in viral infection: Implications across the viral life cycle
Source: Virulence. 2026 Mar 23;17(1):2646692. doi: 10.1080/21505594.2026.2646692 (PMC13034636; doi:10.1080/21505594.2026.2646692)
Supplement: Table S1.docx [file KVIR_A_2646692_SM1885.docx]

| **Table S1. Summary of the contributions of vimentin and keratins to the early stages of viral infection** | | | | | | |
| --- | --- | --- | --- | --- | --- | --- |
| **Virus (type)** | **IF family member** | **Viral factor engaging IF** | **Role in early infection** | **Proposed mechanism** | **Experimental model** | \|  \| \| --- \|  \| **Ref.** \| \| --- \| |
| PRRSV (RNA) | Vimentin (cell surface) | NSP2 | Pro-viral | Vimentin acts as receptor/core component of receptor complex; binds NSP2 to promote adsorption; can confer susceptibility to non-permissive cells | Cell culture models; Piglet model | [32–34] |
| JEV (RNA) | Vimentin (cell surface) | E | Pro-viral | Vimentin serves as key receptor; antibody blockade inhibits infection | Cell culture models | [35,36] |
| JEV (RNA) | Vimentin (cell surface) | — | Pro-viral | D2R-PLC signaling increases cell-surface vimentin, enhancing viral binding and entry | Cell culture models | [37] |
| JEV (RNA) | Vimentin (cell surface) | — | Tropism determinant | Cell-type distribution of vimentin correlates with susceptibility (high in neural progenitor/glia; absent in mature neurons) | Neural cell types/tissues | [38] |
| SARS-CoV (RNA) | Vimentin (cell surface) | Spike | Pro-viral | Spike binds vimentin and promotes its surface accumulation; vimentin blockade/knockdown reduces binding/entry | Cell culture models | [39] |
| SARS-CoV-2 (RNA) | Vimentin (cell surface) | Spike (esp. S1) | Pro-viral | Functions as attachment factor/co-receptor; cooperates with ACE2 to promote entry (incl. endothelial cells) | Cell culture models | [40–43] |
| SARS-CoV-2 (RNA) | Vimentin (cell surface) | Spike | Pro-viral | Interaction may occur at specialized regions (e.g., cilia), organizing local docking platforms for attachment | Cell culture models; primary cilia | [44] |
| DENV-2 (RNA) | Vimentin (cell surface) | E protein EDIII | Pro-viral | EDIII–vimentin binding facilitates adsorption | Cell culture models | [45] |
| CHPV (RNA) | Vimentin (cell surface) | Glycoprotein | Pro-viral | Uses vimentin as co-receptor to promote entry in neuronal cells | Cell culture models | [46] |
| H9N2 AIV (RNA) | Vimentin (cell surface) | HA | Pro-viral | Vimentin required for viral attachment | Cell culture models | [47] |
| EV71 (RNA) | Vimentin (cell surface) | VP1 | Pro-viral | Vimentin required for viral attachment | Cell culture models; mouse model | [48–50] |
| NDV (RNA) | Vimentin (cell surface) | HN | Pro-viral | Vimentin required for viral internalization | Cell culture models | [51] |
| PRV (DNA) | Vimentin (cell surface) | gD, gH | Pro-viral | Viral gD/gH bind conserved sites in vimentin rod domain, facilitating entry | Cell culture models | [52] |
| CMV (DNA) | Vimentin | — | Pro-viral | Vimentin network facilitates entry | Cell culture models | [53,54] |
| HBV (DNA) | Vimentin | — | Pro-viral | \| Required for endocytic entry \| \| --- \|  \|  \| \| --- \| | Cell culture models; woodchuck model | [55] |
| Rotavirus (RNA) | Vimentin (cell surface) | VP4 | Pro-viral | VP4 interacts with vimentin (and ACTR2) to promote early infection | Cell culture models | [56] |
| HPV16 (DNA) | Vimentin (cell surface) | — | Anti-viral | Restricts internalization after attachment, inhibiting infection | \| Cell culture models \| \| --- \|  \|  \| \| --- \| | [57,58] |
| PRV (DNA) | Keratin 1 （KRT1）(cell surface) | — | Pro-viral | KRT1 acts as a key receptor; antibody blockade or KO reduces susceptibility | Cell culture models | [59] |
| Multiple/epithelial context | Keratins (tissue surface) | — | Context-dependent (dual) | Maintain epithelial barrier integrity—may limit tissue invasion while potentially supporting attachment in some settings | Epithelial tissue model | [60] |
