# Supplementary material for: Crosstalk between vimentin and keratins in viral infection: Implications across the viral life cycle
Source: Virulence. 2026 Mar 23;17(1):2646692. doi: 10.1080/21505594.2026.2646692 (PMC13034636; doi:10.1080/21505594.2026.2646692)
Supplement: Table S3.docx [file KVIR_A_2646692_SM1884.docx]

| **Table S3. Summary of the regulatory roles of vimentin in DNA virus infections** | | |  |
| --- | --- | --- | --- |
| **Virus** | **Viral protein** | **Regulatory description** | **Ref.** |
| HBV | HBx | The HBV HBx protein promotes liver cancer development by downregulating LINC01010. | [119] |
|  | - | Vimentin mediates the endocytic entry of HBV. | [55] |
| PCV2 | Cap | Vimentin interacts with the viral Cap protein to inhibit viral replication and particle production by suppressing NF-κB activity and promoting apoptosis. | [81] |
|  | - | PCV2 promotes its own replication by inducing vimentin phosphorylation via CaMKIIγ, which alters its cellular distribution. | [66] |
| VV | P39 | The virus induces vimentin redistribution to the vicinity of replication factories, facilitating viral replication and assembly. | [76] |
| PRV | gD/gH | Vimentin acts as a universal receptor for pseudorabies virus, with viral gD/gH proteins binding its rod domain to facilitate the entire viral life cycle, including viral adsorption, entry, replication, and release. | [52] |
| HBoV1 | VP1u | Vimentin interacts with the HBoV1 VP1u protein, enhancing viral replication and infection by promoting its nuclear transport. | [104] |
| CMV | - | The vimentin network promotes viral entry and transport, and viruses with a broader cell tropism are more dependent on this network. | [53] |
|  | - | An intact vimentin network facilitates viral entry, but the virus subsequently degrades vimentin to complete its replication. | [54] |
| HPV16 | - | Vimentin restricts HPV16 infection by binding viral particles and inhibiting their cellular entry. | [57] |
|  | - | The globular form of vimentin transiently inhibits HPV16 internalization. | [58] |
| ASFV | - | The virus phosphorylates vimentin to form a cage-like structure around replication factories, concentrating viral proteins for efficient assembly. | [106] |
| MVM | - | The vimentin network enhances viral replication by facilitating endosomal transport. | [100] |
| VACV | - | The vimentin network structure facilitates viral replication. | [62] |
