# Supplementary material for: Crosstalk between vimentin and keratins in viral infection: Implications across the viral life cycle
Source: Virulence. 2026 Mar 23;17(1):2646692. doi: 10.1080/21505594.2026.2646692 (PMC13034636; doi:10.1080/21505594.2026.2646692)
Supplement: Table S4.docx [file KVIR_A_2646692_SM1883.docx]

| **Table S4. Overview of keratin-mediated regulation of virus infections** | | | |  |
| --- | --- | --- | --- | --- |
| **Virus type** | **Virus** | **Viral protein** | **Regulatory description** | **Ref.** |
| RNA virus | Influenza virus | NP | IAV induces KRT8 phosphorylation, which in turn promotes its own replication through an interaction with the viral NP protein. | [95] |
|  |  |  | KRT6A binds IAV NP, blocking its interaction with importin α3 to inhibit nuclear import of vRNPs and newly synthesized NP. This also disrupts the vRNP complex, suppressing viral polymerase activity and replication. | [99] |
|  | RSV | GBP5 | KRT9 interacts with RSV GBP5 to promote the secretion of the RSV-SH protein via micro-vesicles, thereby inhibiting viral replication. | [97] |
|  |  | - | KRT18 and its partner KRT8 are essential host factors for RSV replication, supporting both viral genome replication and infectious particle production. | [92] |
|  | LCMV | NP | The LCMV NP protein interacts with KRT1 to stabilize the keratin network, enhancing cell-cell adhesion and promoting its own intercellular spread. | [113] |
|  | HIV/SIV | Capsid/Vpx | KRT72 restricts HIV-1 replication by hindering capsid nuclear transport. However, SIV Vpx counteracts this by binding and degrading KRT72, relieving the restriction and enabling HIV-1 to infect resting CD4+ T cells. | [107] |
|  | HCV | - | KRT8 promotes HCV replication | [93] |
| DNA virus | VZV | ORF62 | The VZV ORF62 protein interacts with and upregulates KRT15 to promote viral replication. | [96] |
|  |  | - | VZV promotes its replication by upregulating the ubiquitin ligase MDM2, which degrades KRT10 and subsequently increases NR4A1 expression. | [98] |
|  | HSV | - | Keratin protects against initial HSV infection but, by shielding the virus from immunity, promotes recurrent lesions at mucocutaneous junctions. | [60] |
|  | HBV | - | KRT8 promotes HBV replication. | [94] |
|  | HPV | - | HPV evades CXCL9/CXCL10-mediated CD8+ T cell clearance by upregulating KRT17. | [117] |
|  | PRV | - | KRT1 is a key receptor for PRV entry. | [59] |
