# Supplementary material for: Crosstalk between vimentin and keratins in viral infection: Implications across the viral life cycle
Source: Virulence. 2026 Mar 23;17(1):2646692. doi: 10.1080/21505594.2026.2646692 (PMC13034636; doi:10.1080/21505594.2026.2646692)
Supplement: Table S2.docx [file KVIR_A_2646692_SM1882.docx]

| **Table S2. Summary of the regulatory roles of vimentin in RNA virus infections** | | |  |
| --- | --- | --- | --- |
| **Virus** | **Viral protein** | **Regulatory description** | **Ref.** |
| Influenza virus | PB2 | IAV enhances replication by upregulating miR-1290 to suppress vimentin, causing nuclear retention of vRNPs. | [91] |
|  | HA | Vimentin promotes the entry and replication of H9N2 AIV through its interaction with the HA protein. | [47] |
|  | - | Vimentin promotes IAV replication by facilitating its endosomal trafficking, acidification, and subsequent genome release. | [103] |
|  | - | Vimentin knockout promotes viral replication by exacerbating the AIV-induced elevation of cholesterol. | [86] |
|  | - | Vimentin enhances IAV membrane fusion and genome release by promoting transport from early to late endosomes. | [102] |
| SARS-CoV | Spike protein | Vimentin acts as a SARS-CoV receptor; its interaction with the spike protein upregulates its surface expression, promoting viral attachment and entry. | [39] |
| SARS-CoV-2 | Spike protein | Vimentin acts as a co-receptor for SARS-CoV-2, interacting with the spike protein and cooperating with ACE2 to mediate viral adsorption and entry. | [40, 42, 43] |
|  |  | Vimentin promotes SARS-CoV-2 entry by interacting with the spike protein, an interaction effectively blocked by the CR3022 antibody. | [41] |
|  |  | The novel recombinant vimentin rod domain binds the viral spike protein with high affinity, blocking its interaction with ACE2 to inhibit replication and attenuate pathogenicity. | [125] |
|  |  | The interaction between vimentin and the spike protein may occur at specific cell surface locations, including ciliary structures, which can serve as docking platforms for SARS-CoV-2. | [44] |
| DENV | NS1 | Disrupting vimentin disassembles its complex with host hnRNPs and viral NS1, inhibiting key protein expression and blocking DENV replication and release. | [109] |
|  | NS4A | The DENV NS4A protein interacts with vimentin, inducing its structural rearrangement to enhance the aggregation of viral replication complexes, thereby promoting viral replication. | [73] |
| DENV-2 | EDIII | The EDIII domain of the DENV-2 envelope protein promotes viral attachment to host cells by interacting with vimentin. | [45] |
|  | - | DENV-2 disrupts vimentin's antiviral function by inducing its structural redistribution. | [67] |
|  | - | DENV-2 activates host ROCK kinase to induce vimentin phosphorylation, promoting its perinuclear rearrangement and thereby facilitating viral replication. | [68] |
|  | - | DENV-2 overcomes vimentin's inhibition in neuronal cells by inducing its phosphorylation and solubilization, promoting its replication and exacerbating pathogenicity in mice. | [89] |
| PRRSV | NSP2 | As a known receptor for PRRSV, cellular vimentin interacts with the viral NSP2 protein to form a complex, thereby synergistically promoting viral attachment and replication. | [33] |
|  | ANXA2/N | The interaction between vimentin and the PRRSV ANXA2 protein facilitates its subsequent binding to the viral N protein, thereby enhancing viral replication. | [72] |
|  | - | As a core component of the viral receptor complex, vimentin not only mediates PRRSV entry but also regulates its intracellular trafficking. | [32] |
|  | - | Vimentin works in concert with other cytoskeletal structures to transport the virus to the perinuclear region. | [101] |
|  | - | PRRSV activates host CaMKIIγ to phosphorylate vimentin, inducing its reorganization into a cage-like structure that encases viral replication complexes to promote viral replication. | [65] |
|  | - | As a receptor for PRRSV, vimentin expression is significantly upregulated following infection. | [34] |
| JEV | E | Vimentin acts as a key receptor for JEV, and its interaction with the viral E protein is crucial for infection; disrupting this binding can effectively block viral entry. | [35] |
|  | NS1, NS1’ | JEV co-opts vimentin via NS1/NS1’-mediated CDK1-PLK1 activation, forming an ER-derived cage to facilitate its replication. | [63] |
|  | - | D2R stimulation enhances JEV infection by upregulating surface vimentin through PLC activation. | [37] |
|  | - | As a JEV receptor, the cell-specific expression of vimentin—high in neural progenitor and glial cells but low in mature neurons—dictates cellular susceptibility and tropism by governing the initial viral binding and entry stage. | [38] |
|  | - | Vimentin acts as a potential receptor for JEV. When fused with an Fc fragment, it can bind JEV particles in vitro, thereby inhibiting viral replication. | [36] |
| HCV | E1 | Vimentin specifically interacts with the viral envelope protein E1 via its N-terminal domain, thereby facilitating efficient cell-to-cell spread of HCV. | [110] |
|  | - | High concentrations of vimentin can inhibit HCV replication by promoting the degradation of the viral core protein. | [80] |
|  | - | Vimentin enhances HCV replication by promoting the proliferation of viral RNA. | [78] |
|  | - | HCV exploits vimentin-rich cellular bridges to directionally transfer viral components from donor cells to adjacent ones, thereby promoting efficient viral spread. | [111] |
| EV | 2A, 3D | An intact vimentin network selectively promotes the synthesis of viral non-structural proteins (2A and 3D), thereby regulating replication and cell survival. | [61] |
| EV71 | VP1 | The EV71 VP1 protein upregulates the expression of its receptor, vimentin, thereby facilitating viral entry and infection. | [48, 49] |
|  | VP1 | The VP1 A289T mutation attenuates EV-71 neurovirulence by weakening its interaction with the vimentin entry receptor. | [50] |
|  | - | EV71 induces the activation of the NLRP3 inflammasome via the VIM-ERK-NF-κB signaling pathway, thereby exacerbating the associated central nervous system damage. | [128] |
| HIV | Gag | M2BP blocks HIV-1 virion production by anchoring Gag to vimentin, preventing its transport to the plasma membrane. | [105] |
|  | - | Vimentin promotes viral replication. | [79] |
|  | - | Vimentin accumulates at virological synapses to promote CD4 clustering, facilitating efficient HIV-1 cell-to-cell spread. | [112] |
| NDV | HN | Vimentin interacts with NDV HN protein to promote viral internalization, replication, membrane fusion, and release, with its rearrangement being particularly crucial for viral replication. | [51] |
|  | - | NDV activates the MLC/p-MLC pathway, inducing vimentin and microfilaments to form cage-like structures that disrupt tight junctions, thereby promoting viral replication and spread. | [64] |
| FMDV | 2C | The FMDV 2C protein assembles a transient, vimentin-based structure to facilitate viral replication. | [74] |
|  | 3A | Vimentin integrity is essential for optimal FMDV replication, as both its intact and compromised states inhibit the process. | [82] |
| RSV | NP | The RSV NP protein interacts with vimentin to induce its degradation and disrupt the cytoskeletal network, thereby promoting virus replication. | [87] |
| BTV | VP2 | The BTV VP2 protein interacts with vimentin to effectively promote viral release. | [108] |
| TGEV | N | The TGEV N protein supports its replication by interacting with vimentin; conversely, disrupting the vimentin network inhibits this process. | [77] |
| ARV | P17 | The ARV p17 protein arrests the cell cycle at G2/M by inhibiting CDK1/Plk1 and disrupting vimentin phosphorylation, creating favorable conditions for viral replication. | [90] |
| CHPV | G | Vimentin acts as a co-receptor by interacting with the CHPV G protein, thereby enhancing the binding of the virus to the cell. | [46] |
| SFTSV | NSs | The NSs protein of SFTSV promotes viral replication by degrading vimentin, which in turn releases the inhibition on autophagy and activates the process. | [88] |
| RV | VP4 | Vimentin interacts with RV VP4 and collaborates with actin-related protein 2 to facilitate early viral infection. |  |
| CSFV | NS5A | CSFV -induced vimentin cages enhance viral replication by recruiting the NS5A protein to the endoplasmic reticulum to stabilize the replication complex. | [56] |
| GAstV-2 | VP70 | Vimentin interacts with the GAstV-2 VP70 protein, promoting its phosphorylation and rearrangement to form an RNA-encapsulating complex that enhances replication. | [71] |
| PRRV | N | Vimentin inhibits viral replication by recruiting the E3 ubiquitin ligase NEDD4L to degrade the viral N protein. | [83] |
| HPIV3 | - | Vimentin inhibits the formation of HPIV3 inclusion bodies by downregulating α-tubulin acetylation through the degradation of α-TAT1. | [84] |
| RHDV | - | RHDV upregulates vimentin, which may facilitate viral spread intracellularly or act as a tissue damage signal upon cell injury. | [126] |
| ZIKV | - | Vimentin encapsulates the ZIKV replication complex to promote viral replication and release, an effect lost upon its disruption. | [69] |
| CHIKV | - | CHIKV exploits the calpain-2-restructured vimentin cage to promote its own replication. | [70] |
| DTMUV | - | DTMUV induces vimentin phosphorylation and rearrangement via CDK5, thereby inhibiting DTMUV replication. | [85] |
| RRV | - | RRV infection alters the cytoskeleton, including vimentin and keratin, which is essential for viral replication and serves as an early sign of damage to intestinal epithelial cells. | [127] |
